# Delayed Diagnosis of Hirschsprung Disease in an 18-Year-Old Male Patient With Fecaloma and Colonic Perforation: A Case Report

**DOI:** 10.7759/cureus.91336

**Published:** 2025-08-31

**Authors:** Alexia Y Martínez Gómez, Salvador Pelayo González, Guillermo Lawers Cuen, Emilio A Chávez Uriarte, Francisco J Ruiz Lara, Mariana Castañeda Llanos, Quitzia L Torres Salazar

**Affiliations:** 1 General Surgery, Hospital General de León, León, MEX; 2 General Surgery, Universidad Autónoma de Guadalajara, Guadalajara, MEX; 3 Biomedical Sciences, Universidad Juárez del Estado de Durango, Durango, MEX

**Keywords:** adolescent megacolon, colonic perforation, delayed diagnosis, fecaloma, hirschsprung disease

## Abstract

Hirschsprung disease is a congenital disorder most commonly diagnosed in the neonatal period or early childhood, typically due to failure to pass meconium within the first 24 hours of life. However, diagnosis may also occur later in adolescence or adulthood in patients with longstanding significant constipation, often leading to delayed recognition and the development of serious complications. We report the case of an 18-year-old male patient with a longstanding history of chronic constipation since infancy, who presented to the emergency department with progressive abdominal distension, abdominal pain, and failure to pass stools. Physical examination revealed severe fecal impaction, and imaging studies demonstrated generalized colonic dilation without evidence of free air. Laboratory findings included hyponatremia, hypokalemia, metabolic acidosis, and evidence of acute decompensation with tissue hypoperfusion. Initial conservative management with intravenous fluids, electrolyte replacement, and enemas provided only partial improvement, and due to progressive deterioration, an urgent exploratory laparotomy was performed. Intraoperative findings revealed megacolon with spontaneous perforation of the descending colon and a large fecaloma. A subtotal colectomy with colostomy was carried out. The diagnosis of short-segment Hirschsprung disease was confirmed histopathologically by the absence of ganglion cells in the submucosal and myenteric plexuses. The postoperative course was favorable, and intestinal transit was later restored in a staged surgical approach. This case highlights the importance of considering organic causes in adolescents with chronic constipation, particularly in the presence of metabolic compromise or acute abdominal complications, as timely surgical intervention can be lifesaving in the context of perforation.

## Introduction

The enteric nervous system (ENS), often referred to as the body's “second brain,” regulates essential gastrointestinal functions, including motility, secretion, and local immune modulation. Among its most clinically significant congenital disorders is Hirschsprung disease (HD), a congenital enteric neuropathy characterized by aganglionosis of the submucosal and myenteric plexuses, leading to functional obstruction of the distal intestine [1]. Its incidence is estimated at approximately one in every 5,000 live births, and most cases are diagnosed during the neonatal period due to the presence of classic symptoms such as failure to pass meconium within the first 24 hours of life, abdominal distension, and bilious vomiting [2].

However, attenuated or short-segment forms may go undetected during early childhood and manifest later in life with chronic constipation, severe fecal impaction, or complications such as megacolon or colonic perforation secondary to severe distension and tissue hypoperfusion. Reports suggest that delayed diagnosis in adolescents and adults accounts for fewer than 5% of cases, often presenting after years of symptoms attributed to functional constipation [3]. In these patients, the diagnostic pitfalls differ from the neonatal form, since chronic constipation is the main expression, but may be overlooked without histopathological confirmation through rectal biopsy.

This report presents the case of an adolescent male patient with a history of significant chronic constipation and recurrent episodes of intestinal obstruction since early childhood, who developed colonic perforation secondary to massive fecal impaction. Chronic constipation in adolescents should not always be assumed to be functional, and HD must remain part of the differential diagnosis.

This case report has been prepared in accordance with the SCARE (Surgical CAse REport) 2025 guidelines [4].

## Case presentation

An 18-year-old male patient with no known history of chronic, surgical, or genetic conditions was brought to the emergency department of a secondary-level general hospital due to progressive abdominal distension, diffuse abdominal pain, nausea, and failure to pass stools for 14 days. The patient reported no episodes of fecal incontinence or soiling. The patient reported similar episodes since early childhood, including at least two previous hospitalizations for suspected intestinal obstruction, though no definitive diagnosis or further investigations had been pursued. No history of fecal incontinence or fecal soiling was reported.

**Figure 1:**
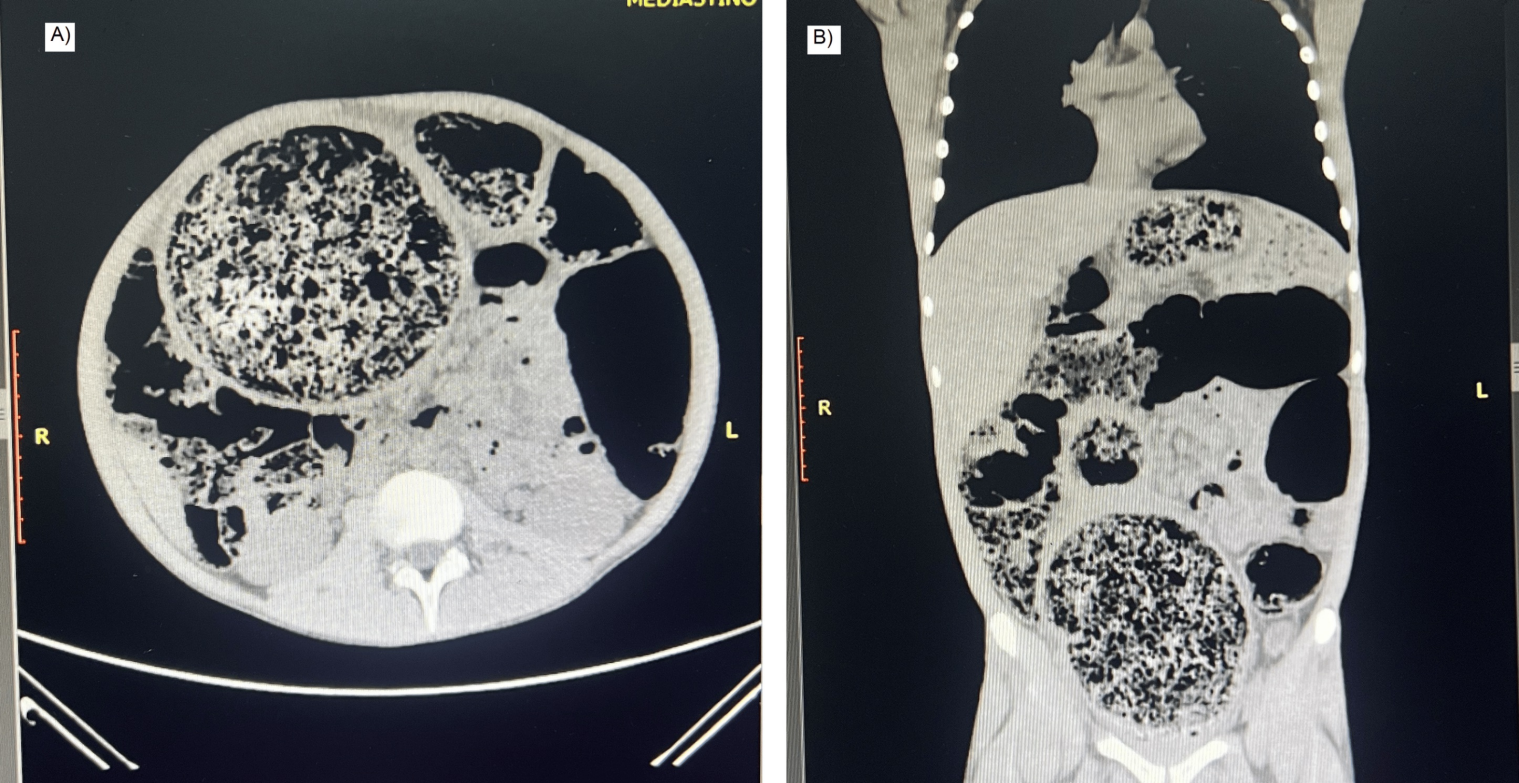
CT of the abdomen showing massive colonic dilation and fecal impaction (A) Axial view demonstrating marked distension of the colon with a large fecaloma. (B) Coronal reconstruction confirming diffuse colonic dilation and extensive fecal material throughout the colon.

**Figure 2:**
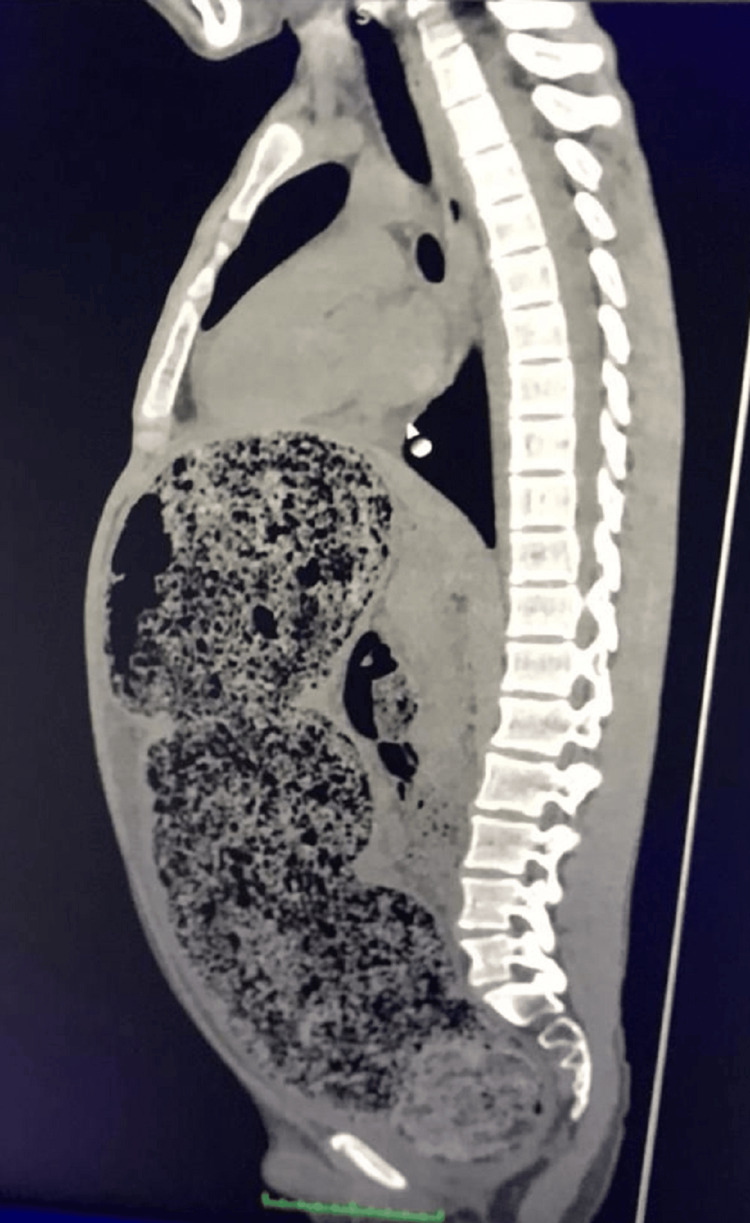
Sagittal CT reconstruction showing marked colonic distension and fecal impaction, consistent with distal intestinal obstruction The compressed diaphragm illustrates the impact of abdominal distension on respiratory compromise.

On physical examination, the patient was somnolent with a heart rate of 90 beats per minute, oxygen saturation of 90%, and marked abdominal distension. Bowel sounds were decreased, and no signs of peritoneal irritation were noted. Digital rectal examination revealed severe fecal impaction with stony consistency, partially removed manually. Abdominal computed tomography (CT) demonstrated massive dilation of the colon with extensive fecal impaction, without evidence of free air (Figure 1). The sagittal reconstruction further confirmed the diffuse colonic distension and the presence of a large fecaloma (Figure 2).

Laboratory analysis revealed hyponatremia (Na⁺ 129 mmol/L), hypochloremia (Cl⁻ 93 mmol/L), hypokalemia (K⁺ 2.8 mmol/L), hypocalcemia (Ca⁺⁺ 1.05 mmol/L), and elevated C-reactive protein (CRP) (16.8 mg/L), indicating systemic inflammation and hydroelectrolytic imbalance (Table 1). No clinical or biochemical evidence of malnutrition was observed. Additionally, progressive metabolic acidosis was documented with decreasing serum bicarbonate (HCO₃⁻ ) (from 20.6 to 18.8 mmol/L), negative base excess (-2.8 to -3.9), and rising lactate levels (from 2.9 to 3.6 mmol/L), reflecting tissue hypoperfusion and metabolic decompensation. Arterial blood gas analysis also revealed a markedly elevated alveolar-arterial gradient (A-aDO₂) from 31 to 83 mmHg and hypoxemia, consistent with impaired gas exchange secondary to respiratory compromise from severe abdominal distension (Table 2). Both mechanisms likely contributed to the patient’s clinical deterioration.

**Table 1:** Serum biochemical parameters at admission NOTE: This table shows alterations in electrolyte balance (hyponatremia, hypokalemia, hypochloremia), inflammatory markers (elevated CRP), and metabolic derangements suggestive of tissue hypoperfusion, supporting the severity of the patient’s clinical condition

Parameter	Patient Result	Unit	Reference Range
Glucose	139.0	mg/dL	74.0 – 106.0
Blood Urea Nitrogen (BUN)	9.3	mg/dL	9.0 – 20.0
Creatinine	0.6	mg/dL	0.66 – 1.25
Uric Acid	3.2	mg/dL	3.5 – 8.5
Amylase	46.0	U/L	30 – 100
Lipase	40.0	U/L	23-300
Phosphorus	2.7	mg/dL	2.5 – 4.5
Lactate	215	U/L	313-618
Chloride	93.0	mmol/L	98.0 – 107.0
Potassium	2.8	mmol/L	3.5 – 5.1
Sodium	129.0	mmol/L	137.0 – 145.0
Magnesium	2.3	mg/dL	1.6 – 2.3
C-Reactive Protein (CRP)	16.8	mg/L	<10
Leukocytes	7.43	10^3^/μL	4-10

**Table 2:** Arterial blood gas parameters at admission and after 24 hours Progressive metabolic acidosis, rising lactate, and worsening alveolar-arterial gradient (A–aDO₂) reflect tissue hypoperfusion and impaired gas exchange, supporting the decision for urgent surgical intervention. pCO2: partial pressure of carbon dioxide; pO2: partial pressure of oxygen; Na+: sodium; K+: potassium; HCO3-: bicarbonate; SO2: oxygen saturation; Ca++: calcium; TCO2: total carbon dioxide; A-aDO2: alveolar-arterial oxygen difference

Parameter	At Admission	At 24 hours after admission	Reference Range
pH	7.43	7.45	7.35 – 7.45
pCO2	31	27	35 – 45 mmHg
pO2	52	82	80 – 100 mmHg
Na+	135	134	135 – 145 mmol/L
K+	3.2	2.8	3.5 – 5.0 mmol/L
Lactate	2.9	3.6	˂ 2 mmol/L
HCO3-	20.6	18.8	22 – 26 mmol/L
Base excess	-2.8	-3.9	-2 to +2
SO2	87	97	95 – 100%
Ca++	1.06	1.05	1.12 – 1.32 mmol/L
TCO2	21.6	19.6	23 – 27 mmol/L
A-aDO2	31	83	<15 mmHg (21% FiO2)

Due to a lack of clinical improvement after 24 hours of conservative management (including intravenous hydration, electrolyte replacement, cleansing enemas, partial manual disimpaction, and analgesia) and in view of worsening biochemical and gasometric parameters, an emergency exploratory laparotomy was performed.

**Figure 3:**
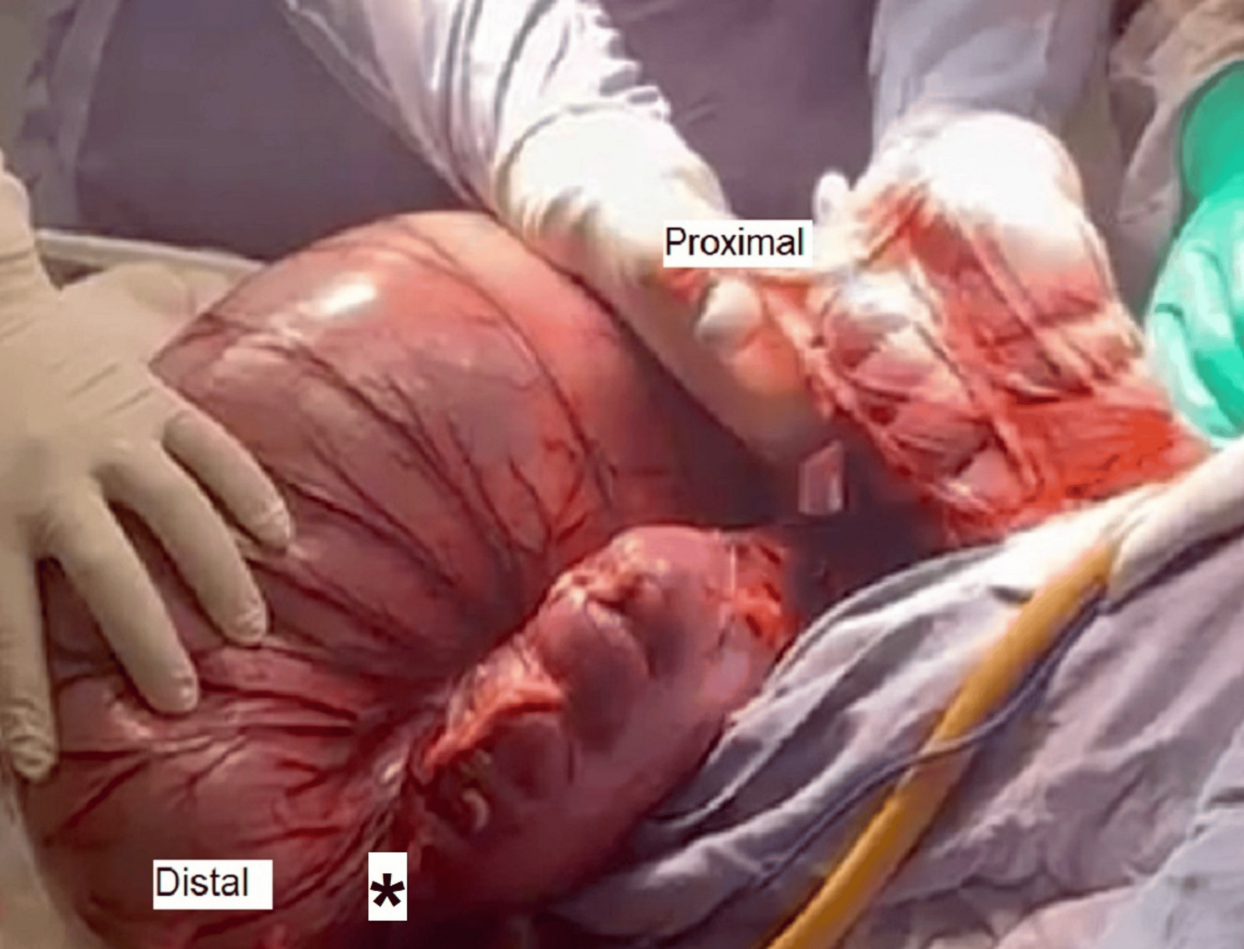
Intraoperative view showing severe colonic dilatation with spontaneous perforation of the descending colon

**Figure 4:**
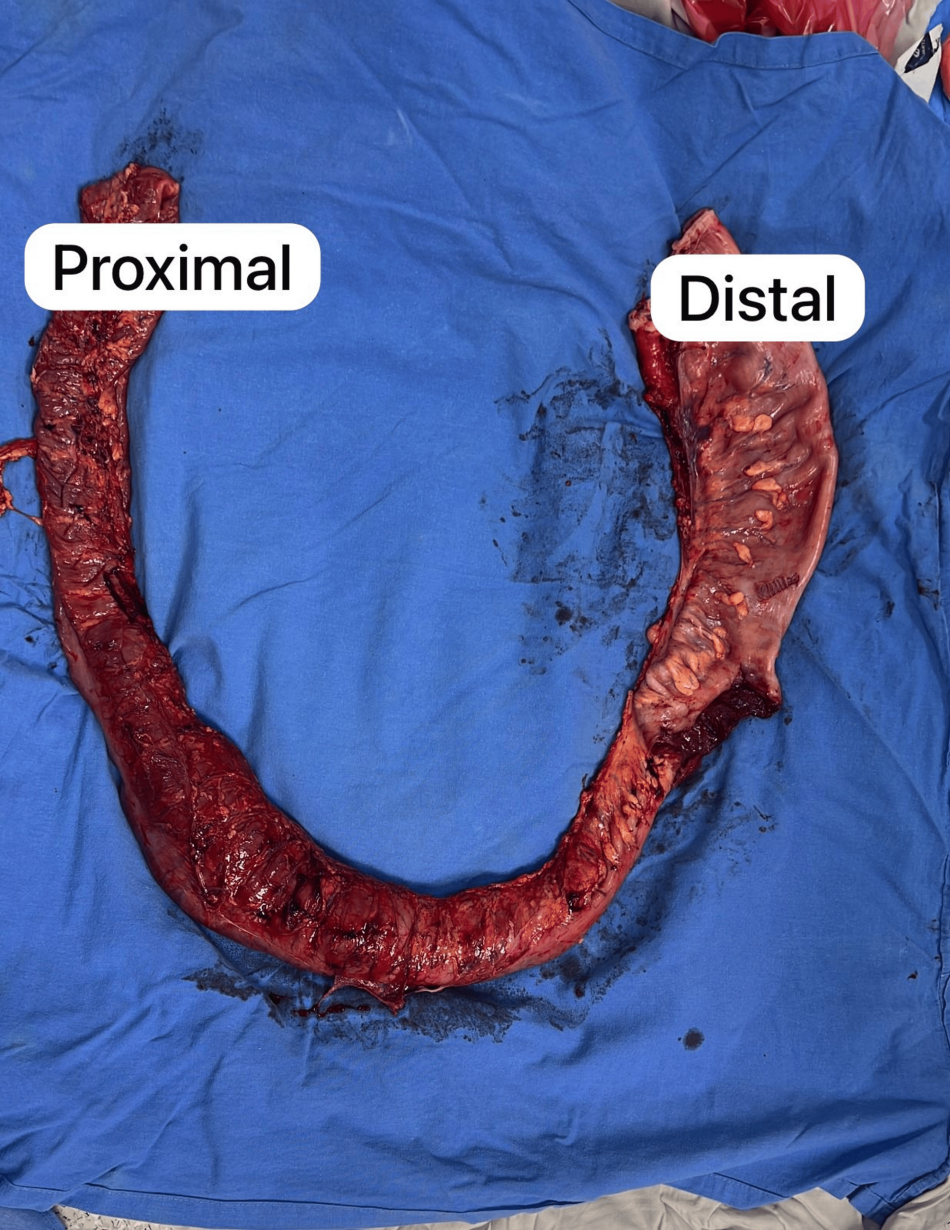
Resected colonic segment showing marked dilatation of the distal colon and abrupt transition to a narrowed proximal segment

Intraoperative findings revealed severe dilation of the ascending and transverse colon (up to 15 cm in diameter), and even more pronounced dilation of the descending and sigmoid colon (up to 30 cm), with displacement of the sigmoid toward the midline and increased intra-abdominal pressure (Figure 3). During surgical manipulation, a 2 cm perforation was observed at the junction of the descending and sigmoid colon, with the release of approximately 10 kg of liquid fecal content. Stony fecal impaction was also found in the mid and upper rectum, with a maximum diameter of 15 cm. The small intestine (approximately 330 cm) appeared unremarkable (Figure 4).

**Figure 5:**
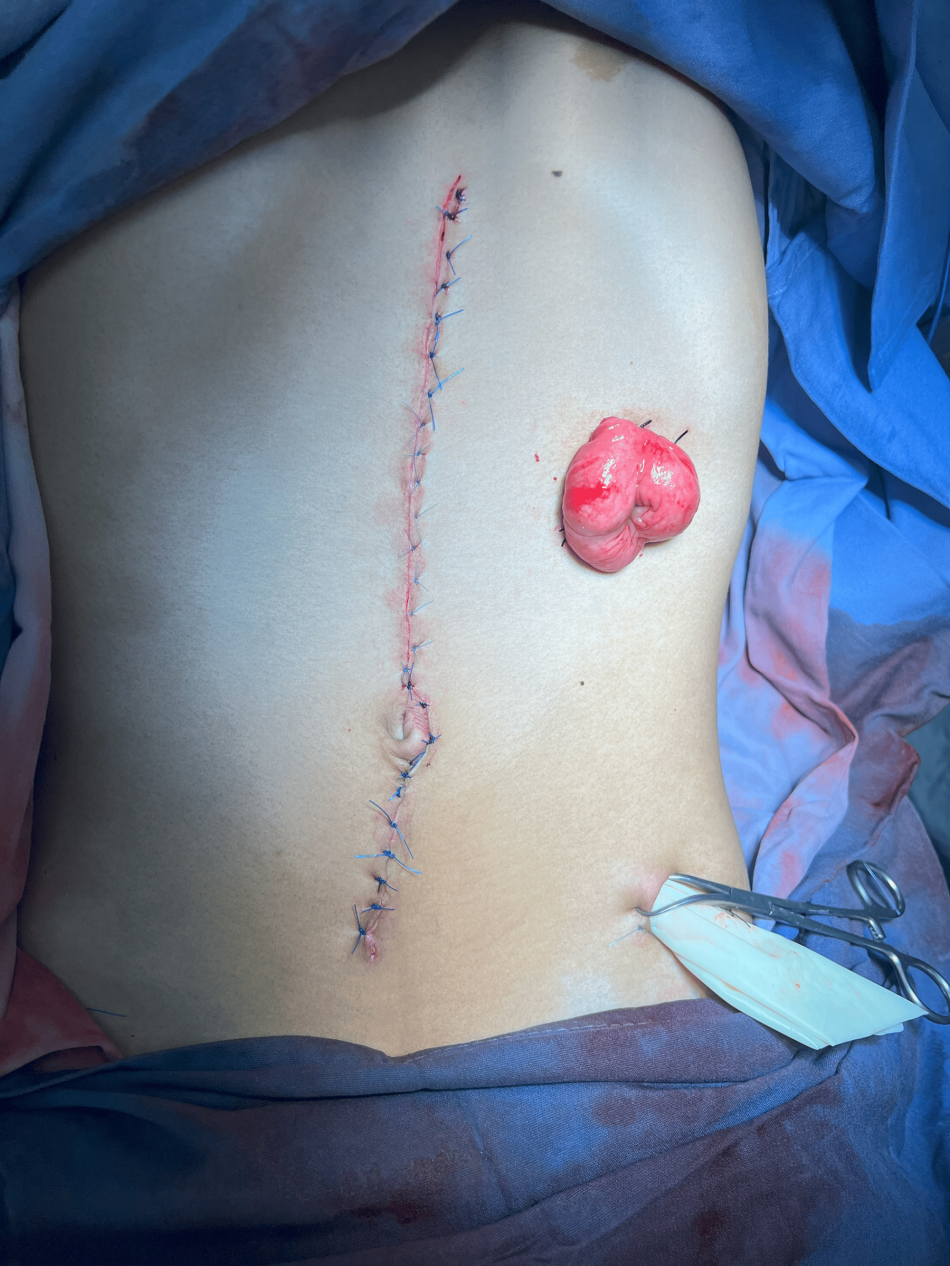
Immediate postoperative view showing the midline laparotomy closure and terminal colostomy exteriorized in the left lower quadrant; a pelvic drain is also observed in situ

Given the emergent setting, a subtotal colectomy (descending and sigmoid colon) was performed, with end colostomy using the transverse colon, closure of the distal stump at the level of the peritoneal reflection, peritoneal lavage, and placement of pelvic drains (Figure 5). This represented the first stage of a planned two-step approach, focused on resolving the obstruction and controlling the perforation. The patient’s postoperative course was favorable, with no immediate complications, and he was discharged home on postoperative day seven, hemodynamically stable.

**Figure 6:**
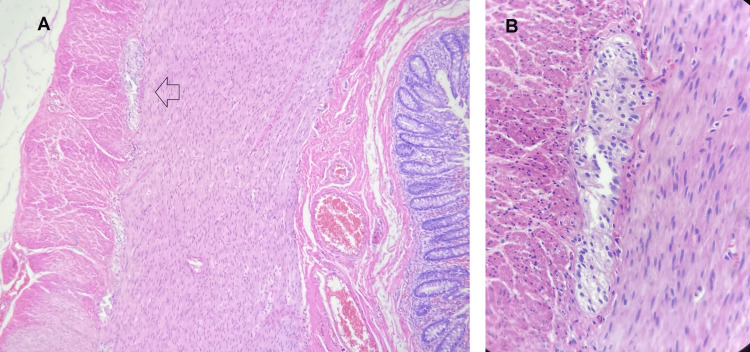
Histopathological examination of the rectosigmoid specimen stained with hematoxylin and eosin (H&E) (A) Section at ×20 magnification showing mucosal ulceration, necrosis, and absence of ganglion cells in both the submucosal (Meissner’s) and myenteric (Auerbach’s) plexuses. (B) Section at ×40 magnification of the submucosa demonstrating a hypertrophic nerve trunk (arrow), consistent with Hirschsprung disease.

**Figure 7:**
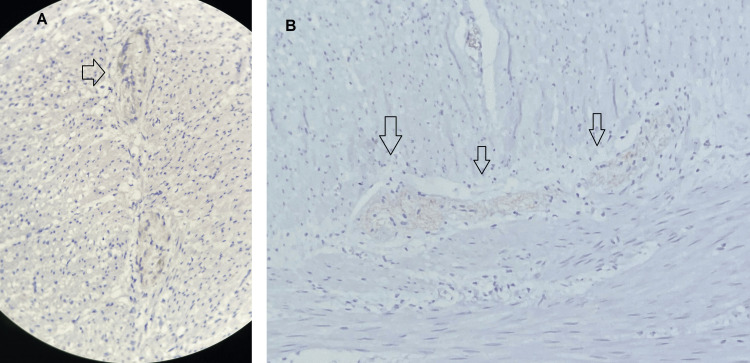
Immunohistochemical staining for calretinin in rectosigmoid specimen (A) Section at ×20 magnification showing absence of calretinin-positive ganglion cells and hypertrophic nerve trunk (arrow). (B) Section at ×40 magnification highlighting multiple hypertrophic nerve trunks in the submucosa (arrows), consistent with aganglionic segment of Hirschsprung disease.

Histopathological examination of the surgical specimen revealed mucosal ulceration, necrosis, colonic perforation, and complete absence of ganglion cells in both the submucosal and myenteric plexuses, confirming the diagnosis of short-segment Hirschsprung disease with a transition zone of approximately 15 cm (Figure 6). Immunohistochemical staining for calretinin further supported these findings, demonstrating absence of calretinin-positive ganglion cells and highlighting the presence of hypertrophic submucosal nerve trunks (Figure 7). Six months later, a second procedure was performed to restore intestinal continuity, which was completed without complications and resulted in favorable clinical progress with definitive discharge from surgical follow-up. 

## Discussion

This case highlights an uncommon presentation of HD, characterized by delayed diagnosis during adolescence and severe complications such as massive fecal impaction and colonic perforation secondary to severe distension. In contrast, the classic neonatal form typically manifests with failure to pass meconium within the first 24 hours of life, abdominal distension, and bilious vomiting. As reported by Rojas-Gutiérrez et al., HD in young adults may emerge in a more insidious and progressive fashion, often presenting as significant chronic constipation punctuated by acute decompensations, whether due to sigmoid volvulus or, as in our case, colonic perforation related to fecaloma [5].

The underlying pathophysiology in both early and late forms stems from aganglionosis of the submucosal and myenteric plexuses, a consequence of defective neural crest cell migration and differentiation during embryogenesis. Klein et al. emphasize that this defect classifies HD among the neurocristopathies, with its clinical expression shaped by both the length of the aganglionic segment and the adaptive capacity of the proximal bowel [6]. This variability helps explain why some patients, like ours, may reach adolescence undiagnosed despite longstanding symptoms.

Late-diagnosed HD poses a diagnostic challenge because, although chronic constipation is a cardinal clinical expression, in older patients it is often attributed to functional disorders, delaying a thorough etiologic evaluation. In our case, despite a long-standing history of significant chronic constipation since infancy, the definitive diagnosis was established only after an acute surgical complication prompted resection and histopathological examination. This scenario mirrors the experience described by Soussan et al., who reported a 20-year-old with long-standing constipation in whom an episode of acute intestinal obstruction triggered the diagnostic work-up that confirmed HD, underscoring the risk of underdiagnosis into adulthood [7].

CT revealed marked colonic dilatation with an abrupt transition zone suggestive of an aganglionic segment. Operative findings included a markedly dilated colon with a narrowed rectum, a feature that should raise suspicion for short- or ultra-short-segment HD and help distinguish it from functional constipation, where the rectum is usually dilated. Although diagnostic tools such as anorectal manometry and endoscopic evaluation may be helpful, they can fail to provide conclusive results. A rectal biopsy including the submucosa remains the gold standard for diagnosis, and in both our patient and that of Soussan et al. [7], only histopathological assessment of the surgical specimen ultimately confirmed the absence of ganglion cells and established the diagnosis. In our case, immunohistochemical staining for calretinin further confirmed these findings, demonstrating the absence of calretinin-positive ganglion cells and the presence of hypertrophic nerve trunks within the aganglionic segment, which strengthened the diagnostic certainty.

Unlike the case reported by Rojas-Gutiérrez [5], in which electrolyte and metabolic profiles were largely unremarkable, our patient demonstrated pronounced disturbances, including hyponatremia, hypokalemia, metabolic acidosis, and elevated serum lactate, consistent with tissue hypoperfusion. These laboratory abnormalities signaled systemic compromise and served as severity markers prompting timely surgical intervention.

From a pathophysiologic standpoint, the absence of ganglion cells in Auerbach’s and Meissner’s plexuses disrupts coordinated peristalsis, leading to functional obstruction and subsequent megacolon. The underlying defect in neuroblast migration occurs between the fourth and seventh week of gestation, and previous studies have suggested that the length of the aganglionic segment may influence the age and severity of clinical presentation [7].

In terms of surgical management, cases previously reported share a radical approach, subtotal colectomy with fecal diversion followed by a planned intestinal transit reconstruction [5,7]. Our case required urgent surgical intervention due to progressive clinical deterioration and suspected perforation. This underscores the need to individualize surgical decisions, tailoring them to the patient’s presentation, resource availability, and the surgical team’s expertise. 

Several operative approaches have been described in adolescents and adults with Hirschsprung disease, including the Swenson procedure (full-thickness resection and coloanal anastomosis), the Soave technique (endorectal pull-through with preservation of the rectal muscular cuff), and the Duhamel operation (retrorectal side-to-side pull-through). While laparoscopic and transanal techniques have gained popularity in pediatric populations, their role in adults is less well established, and open approaches remain common due to advanced colonic dilatation and fibrosis at presentation. Postoperative outcomes in adults are generally favorable when diagnosis and treatment are not excessively delayed, though complications such as persistent constipation, incontinence, and enterocolitis may occur, underscoring the importance of individualized surgical planning and long-term follow-up [8,9].

## Conclusions

This case highlights the importance of maintaining a high index of suspicion for HD in adolescents with severe chronic constipation and symptoms dating back to early childhood. In such patients, HD must be actively ruled out through a thorough diagnostic evaluation, including rectal biopsy with histopathological confirmation. The distinctive features of our case, such as delayed diagnosis into adolescence, severe fecal impaction with metabolic compromise, and intraoperative identification of colonic perforation, underline the risks of late recognition. The integration of clinical, radiological, and biochemical findings facilitated timely surgical intervention, which was lifesaving and followed by a favorable recovery. These findings emphasize the need for a prompt, multidisciplinary approach that addresses both acute management and long-term functional follow-up planning.

## References

[1] Niesler B, Kuerten S, Demir IE, Schäfer KH (2021). Disorders of the enteric nervous system - a holistic view. Nat Rev Gastroenterol Hepatol.

[2] Joseph S, Guinot A, Leclair M (2019). Hirschsprung's disease [Article in Spanish]. EMC Pediatría.

[3] Atamanalp SS, Peksoz R, Disci E (2024). Comments on "sigmoid volvulus in a young adult, a manifestation of Hirschsprung disease" [Article in Spanish]. Cir Cir.

[4] Kerwan A, Al-Jabir A, Mathew G (2025). Revised Surgical CAse REport (SCARE) guideline: an update for the age of Artificial Intelligence. Premier J Science.

[5] Rojas-Gutiérrez CD, Haro-Cruz JS, Cabrera-Eraso DF, Torres-García VM, Salas-Álvarez JC, Valencia-Jiménez JO (2022). Case report: sigmoid volvulus in a young adult, a manifestation of Hirschsprung disease [Article in Spanish]. Cir Cir.

[6] Klein M, Varga I (2020). Hirschsprung's disease-recent understanding of embryonic aspects, etiopathogenesis and future treatment avenues. Medicina (Kaunas).

[7] Soussan H, Jabi R, Ouryemchi M, Haddadi Z, Bouziane M (2021). Hirschsprung's disease in adults revealed by an occlusive syndrome. Cureus.

[8] Nguyen BU, Vu MT, Pham QT (2023). Adopting the Swenson-like technique for patients with Hirschsprung disease in Vietnam. Pediatr Surg Int.

[9] Montalva L, Cheng LS, Kapur R (2023). Hirschsprung disease. Nat Rev Dis Primers.

